# Lung epithelial cell-derived extracellular vesicles activate macrophage-mediated inflammatory responses via ROCK1 pathway

**DOI:** 10.1038/cddis.2015.282

**Published:** 2015-12-10

**Authors:** H-G Moon, Y Cao, J Yang, J H Lee, H S Choi, Y Jin

**Affiliations:** 1 Division of Pulmonary and Critical Care, Department of Medicine, Brigham and Women's Hospital, Harvard Medical School, 75 Francis Street, Boston, MA 02115, USA; 2 Division of Hematology/Oncology, Department of Medicine, Beth Israel Deaconess Medical Center, Boston, MA 02215, USA

## Abstract

Despite decades of research, the pathogenesis of acute respiratory distress syndrome (ARDS) remains poorly understood, thus impeding the development of effective treatment. Diffuse alveolar damage (DAD) and lung epithelial cell death are prominent features of ARDS. Lung epithelial cells are the first line of defense after inhaled stimuli, such as in the case of hyperoxia. We hypothesized that lung epithelial cells release ‘messenger' or signaling molecules to adjacent or distant macrophages, thereby initiating or propagating inflammatory responses after noxious insult. We found that, after hyperoxia, a large amount of extracellular vesicles (EVs) were generated and released into bronchoalveolar lavage fluid (BALF). These hyperoxia-induced EVs were mainly derived from live lung epithelial cells as the result of hyperoxia-associated endoplasmic reticulum (ER) stress. These EVs were remarkably different from epithelial ‘apoptotic bodies', as reflected by the significantly smaller size and differentially expressed protein markers. These EVs fall mainly in the size range of the exosomes and smaller microvesicles (MVs) (50–120 nm). The commonly featured protein markers of apoptotic bodies were not found in these EVs. Treating alveolar macrophages with hyperoxia-induced, epithelial cell-derived EVs led to an increased secretion of pro-inflammatory cytokines and macrophage inflammatory protein 2 (MIP-2). Robustly increased macrophage and neutrophil influx was found in the lung tissue of the mice intranasally treated with hyperoxia-induced EVs. It was determined that EV-encapsulated caspase-3 was largely responsible for the alveolar macrophage activation *via* the ROCK1 pathway. Caspase-3-deficient EVs induced less cytokine/MIP-2 release, reduced cell counts in BALF, less neutrophil infiltration and less inflammation in lung parenchyma, both *in vitro* and *in vivo*. Furthermore, the serum circulating EVs were increased and mainly derived from lung epithelial cells after hyperoxia exposure. These circulating EVs also activated systemic macrophages other than the alveolar ones. Collectively, the results show that hyperoxia-induced, lung epithelial cell-derived and caspase-3 enriched EVs activate macrophages and mediate the inflammatory lung responses involved in lung injury.

Acute lung injury (ALI) and its severe form, ARDS cause significant morbidity and mortality in critically-ill patients.^1^ ALI often presents with extensive accumulation of activated inflammatory cells and diffuse alveolar damage (DAD) accompanied by oxidative stress.^2^ Lung epithelial cell damage, a prominent feature of both infectious and non-infectious lung injury, potentially has an important functional role in the pathogenesis of the overwhelming inflammation and vascular leaking involved in ALI/ARDS.^3, 4^ However, it remains incompletely understood how lung inflammation is initiated and propagated during the development of lung injury, particularly by non-infectious stimuli. For example, oxidative stress, such as occurs with the inspiration of a high concentration of oxygen, could lead to reactive oxygen species (ROS) production, inflammasome activation, pro-inflammatory cytokine production, neutrophil influx and lung inflammation,^5, 6^ resulting in severe lung injury and respiratory failure. It has been reported that the deposition of extracellular matrix (ECM) has a role in this process.^7^ Therefore, the cross-talk between damaged epithelial cells and lung inflammation cells during the development of non-infectious lung injury needs to be explored to properly understand the development of ALI/ARDS.

Hyperoxia-induced ALI (HALI) is a well-established, non-infectious animal model that mimics human ARDS and has been used extensively by investigators to better understand the pathogenesis of this devastating syndrome.^8^ Oxidative stress, such as occurs with hyperoxia and its derivative ROS, can induce epithelial cell death *via* apoptosis, autophagic cell death, necrosis and many other pathways.^9^ Prolonged exposure to a high concentration of oxygen is fatal in most animal models, resulting in neutrophil influx and alveolar edema.^6^ However, despite the fact that mouse HALI is a good model of human ARDS, mortality in rodents often results from severe cerebral edema.^6^ Activated alveolar macrophage-released chemokines/cytokines are essential to neutrophil recruitment.^6^ That said, how the oxidative stress specifically activates alveolar macrophages has not been well elucidated. In this study, we used the mouse model of HALI to evaluate the cross-talk between damaged lung epithelial cells and alveolar macrophages during the development of HALI *via* epithelial cell-derived EVs.

For a long time, EVs were considered membrane debris without any specific biological function.^10^ Recently, accumulating data have suggested that EVs are in fact crucial mediators of intercellular communications.^11, 12, 13^ EVs are categorized into exosomes, microvesicles and apoptotic bodies based on their origin, size and content.^10^ The exosome is 40–120 nm in size and is originated from the endo-lysosomal pathway, intraluminal budding or the fusion of multivesicular bodies with the cell membrane. It is characterized by holding plasma membrane proteins such as the tetraspanin (CD9, CD63, CD81 and so on) and lipid raft proteins (flotillin and caveolin-1).^14^ The exosome also contains mRNA and microRNA (miRNA) as well as cytoplasmic and membrane proteins. It is secreted from majority of cells, including macrophages, dendritic cells and epithelial cells among many others. Microvesicles (MVs) are 50–1000 nm in size and are originated from the outward budding of the cell membrane.^10^ MVs contain membrane proteins, mRNA, miRNA, non-coding RNAs and cytoplasmic proteins.^10^ Apoptotic bodies are significantly larger than exosomes and MVs, averaging 500–2000 nm, and are generated from the surface of apoptotic cells.^10^ They are characterized by a large amount of phosphatidylserine, cell organelles, nuclear fractions and certain marker proteins, such as Apaf-1.^10^ Both infection and toxic insults have been reported to facilitate the generation of EVs.^15, 16, 17^ EVs are reported to have similar cellular functions as their mother cells.^10, 18^ For instance, resting macrophage-originated MVs exert an anti-inflammatory effect, whereas macrophage-originated MVs are pro-inflammatory after LPS stimulation.^19^ Although EVs appear promising candidates for intercellular communication, their roles in lung cells, particularly in the pathogenesis of ALI, have not been reported.

We hypothesized that hyperoxia-associated oxidative stress stimulates EV generation in lung epithelial cell and that epithelial cell-derived EVs facilitate the development of inflammatory lung responses after oxidative stress. We further explored the components in epithelial cell-derived EVs after hyperoxia. The underlying mechanisms by which EVs exert their pro-inflammatory effects on alveolar macrophages were also determined. To the best of our knowledge, this is the first study focusing on the role of EVs in the pathogenesis of hyperoxia-induced ALI, the intercellular cross-talk between epithelial cells and alveolar macrophages, as well as the relationship between cell death and pro-inflammatory signals.

## Results

### Hyperoxia stimulated the production of EVs in lung epithelial cells

**Figure 1:**
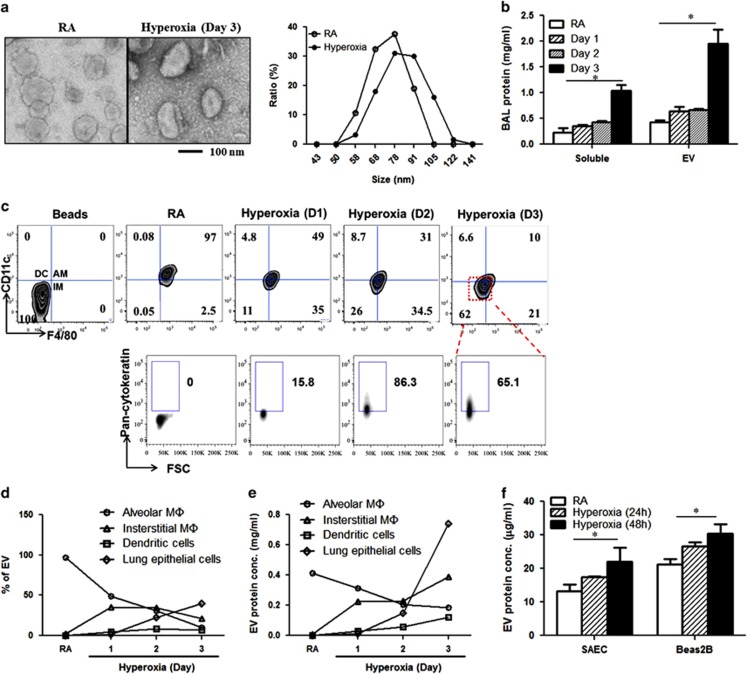
Hyperoxic stress promoted EV production *in vivo* and *in vitro*. Mice were exposed to hyperoxia (100% O_2_) for the designated time period; BALF EVs were isolated using serial centrifugation (1000  × *g*→10 000 × * g*→100 000 × *g*). (**a**) Negative stained transmission electron microscopy (TEM) image (left panel). The size of BALF EVs was measured by DLS (right panel) after 3 days' hyperoxia. (**b**) Protein concentrations in BALF EVs and soluble fractions. (**c**) The origin of BALF EVs. CD11c^+^F4/80^−^ stands for dendritic cells, CD11c^−^F4/80^+^ for interstitial macrophages, CD11c^+^F4/80^+^ for alveolar macrophages (upper) and pan-cytokeratin for epithelial cells (lower). (**d**) Dynamic changes on the percentages of cell type-specific EVs in BALF after hyperoxia. (**e**) Dynamic changes on the EV protein levels in each cell type after hyperoxia. (**f**) EV protein levels in the supernatant of small airway epithelial cells (SAECs) and bronchial epithelial cells (Beas2B), after hyperoxia. Error bars represent mean±S.D. *n*=3. All other figures represent two independent experiments with identical results. *P<0.05

To determine the cross-talk that takes place between lung epithelial cells and alveolar macrophages after hyperoxic stress, we first assessed whether hyperoxia stimulates EV generation from lung epithelial cells by following previously described.^20^ After exposure to hyperoxia (1–3 days), we isolated EVs by exploiting serial centrifugation (1000 × *g*→10 000 × *g*→100 000 × *g*), and EVs were confirmed in mouse BAL fluid (BALF) using TEM imaging (Figure 1a, left). The size of these BALF EVs was analyzed using dynamic light scattering (DLS). We found that the size ranged from 50 to 120 nm (Figure 1a, right). The expression patterns of Apaf-1 (a marker of apoptotic bodies), CD40L and integrin *β*1 (markers of MVs), Vps27, Vps32, Vps24, Vps4 and flot-1 (markers for exosomes) were also analyzed in these EVs (Supplementary Figure S1A).^10^ Room air (RA)-associated EVs expressed the exosome markers and hyperoxia-induced EVs carried the markers for both MVs and exosomes (Supplementary Figure S1A). Neither RA-associated EVs nor hyperoxia-induced EVs expressed Apaf-1 (Supplementary Figure S1A), suggesting that these EVs were different from apoptotic bodies.^10^ Moreover, we measured the cholesterol level in the BALF EVs from mice (Supplementary Figure S1B). Furthermore, the level of mitochondrial DNA (mtDNA) in these EVs was not changed after hyperoxia (Supplementary Figure S1C). On the basis of their size, these hyperoxia-induced EVs fit in the range of exosomes to MVs and were markedly smaller than apoptotic bodies. The BALF EV protein levels were also significantly increased in a time-dependent manner in hyperoxia (Figure 1b). To determine which cells are responsible for the increased EV production after hyperoxia, we analyzed the cellular markers expressed in the BALF EVs using FACS. The majority of the hyperoxia-induced BALF EVs was originated from non-mononuclear cells (>62%), rather than from alveolar macrophages (10%), interstitial macrophages (21%) or dendritic cells (6.6%) (Figure 1c). Interestingly, in the case of RA, alveolar macrophages were primarily responsible for the production of EVs in BALF. However, lung epithelial cell-generated EVs were dramatically increased after hyperoxia in a time-dependent manner, as demonstrated by the amount of both the EVs and EV proteins, as shown in Figures 1d and e, respectively. On the other hand, after hyperoxia, the percentages of EVs derived from alveolar macrophages, interstitial macrophages and/or dendritic cells were either decreased or blunted (Figures 1d and e). To confirm that lung epithelial cells indeed generate large amounts of EVs after hyperoxia, using both human small airway epithelial cells (SAECs) and human bronchial epithelial cells (Beas2B) (Figure 1f).

### ER stress-associated signaling pathways mediated hyperoxia-induced EV production and secretion in lung epithelial cells

**Figure 2:**
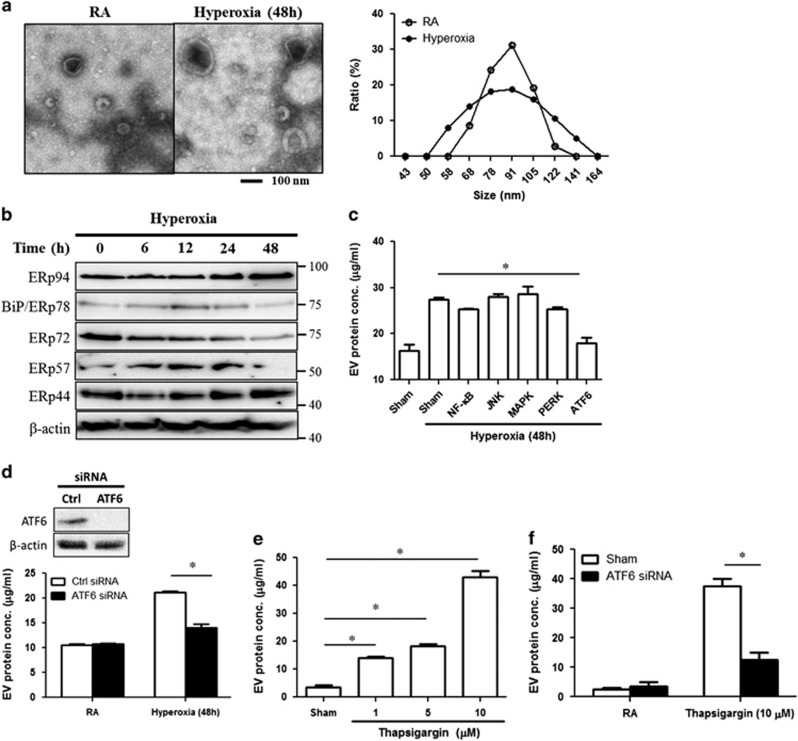
Hyperoxia-induced ER stress stimulated EV secretion from lung epithelial cells. (**a**) Beas2B cells were exposed to hyperoxia (95% O_2_, 5% CO_2_). After 48 h, EV was isolated from conditioned media using serial centrifugation. Negative stained transmission electron microscopy (TEM) image (left panel) and size of Beas2B EVs measured by DLS (right panel). (**b**) After 6–48 h, the expressions of ER stress-related proteins were analyzed. (**c**) Beas2B cells were exposed to hyperoxia in the presence of pathway inhibitors (10 *μ*M/sample). After 48 h, EVs were isolated and protein concentration was measured. Bay11-7082 for NF-*κ*B, JNK II inhibitor for JNK, SB 203580 for p38 MAPK, Perk inhibitor for Perk, *γ*-secretase inhibitor for ATF6 signaling. (**d**) EV production was measured in ATF6 siRNA-transfected Beas2B cells, after hyperoxia (48 h). (**e**) Beas2B cells were treated with ER stress inducer, thapsigargin. After 24 h, EVs were isolated. (**f**) EV production was measured in the ATF6 siRNA-transfected Beas2B cells, 24 h after thapsigargin. Error bars represent mean±S.D. *n*=3. All other figures represent two independent experiments with identical results. *P<0.05

As shown in a previous report, ER stress is an essential factor involved in the damage of lung epithelial cells after hyperoxia.^21^ To determine the underlying mechanisms by which hyperoxia induces EV production in lung epithelial cells, we first treated Beas2B human epithelial cells with hyperoxia (95%) in a time-dependent manner. Beas2B-originated EVs were isolated using serial centrifugation (1000 × *g*→10 000 × *g*→100 000 × *g*), and the purity of EVs was confirmed using TEM and DLS (Figure 2a). We measured ER stress-related proteins in Beas2B whole-cell lysate. The expression of these proteins clearly modulated after hyperoxia (Figure 2b). Using specific ER-signaling inhibitors, we showed that the hyperoxia-induced EV production was suppressed by an ATF6 inhibitor (Figure 2c). This observation was further confirmed using ATF6 siRNA (Figure 2d). The efficacy of ATF6 siRNA was confirmed before these experiments (data not shown). To directly confirm whether ER stress is involved in the augmented production of EVs in Beas2B cells, we treated Beas2B cells with an ER stress inducer, thapsigargin. EV production was highly induced in a dose-dependent manner in these cells after thapsigargin treatment (Figure 2e). Again, pretreatment of these cells with ATF6 siRNA resulted in significantly less EV generation after thapsigargin (Figure 2f).

### Robustly increased caspase-3 in hyperoxia-induced EVs

**Figure 3:**
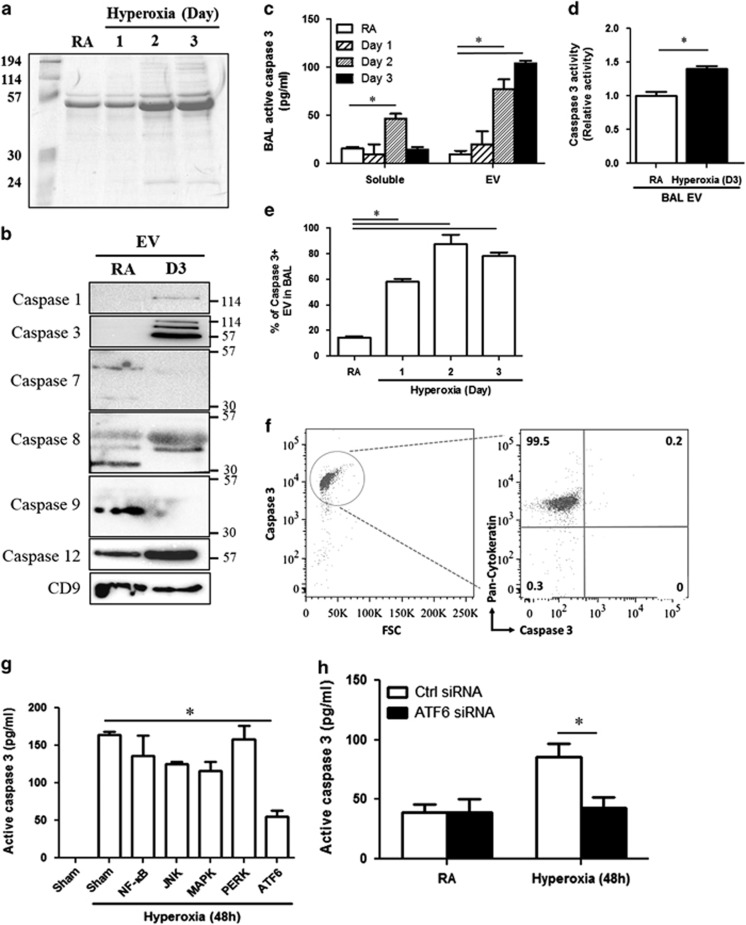
Caspase-3 was enriched in EVs after hyperoxia. (**a**) Hyperoxia-induced, BALF EV proteins (20 *μ*g) were fractioned using 10% SDS-PAGE gel and stained with Coomassie Blue. (**b**) Expression of caspase proteins in BALF EVs after 3 days' hyperoxia exposure. (**c**) Quantification of active caspase-3 in BALF EVs and soluble fractions. Active caspase-3 was measured using ELISA. (**d**) Caspase-3 activity was measured in BALF EVs. (**e**) Percentage of caspase-3-positive BALF EVs after hyperoxia. (**f**) Origins of the caspase-3-positive EVs isolated from BALF. Mice were exposed to 2 days' hyperoxia. Pan-cytokeratin stands for epithelial cells and F4/80 for macrophages. (**g**) Beas2B cells were treated with or without ER stress inhibitors (10 *μ*M), followed by hyperoxia. After 48 h, supernatant EVs were isolated and analyzed for active caspase-3. (**h**) Supernatant EVs were obtained from the control or ATF6 siRNA-transfected Beas2B cells, after hyperoxia (48 h). Error bars represent mean±S.D. *n*=3. All other figures represent two independent experiments with identical results. *P<0.05

To determine the functions of the hyperoxia-induced EVs, we attempted to identify the key components that were encapsulated in these epithelial cell-derived vesicles after hyperoxia. We first compared the total protein levels in the EVs, which were isolated from the cell supernatant after RA or hyperoxia. The levels of the EV proteins were robustly increased after hyperoxia in a time-dependent manner (Figure 3a). Caspases are essential cell components involved in hyperoxia-induced cell damage and death.^22^ The EV-shuttled caspase-3 complex was highly induced after hyperoxia (Figure 3b). The caspase-3 mRNA level exhibited no significant change between the RA-EVs and hyperoxia-EVs (data not shown). Along with caspase-3, caspases-1, -8 and -12 were also augmented in hyperoxia-induced EVs. Caspases-7 and -9 were not found (Figure 3b). The detected size of the EV-shuttled caspase-3 was consistent with a tetramer form of the cleaved fragments (58 KD).^23, 24^ Using ELISA, we measured the active caspase-3 level (cleaved caspase-3) in both the EVs and the soluble fractions of BALF. The active caspase-3 level in EVs was significantly elevated after hyperoxia in a time-dependent manner (Figure 3c). We further confirmed that caspase-3 activity was also enhanced in hyperoxia-induced EVs compared with the EVs generated in RA (Figure 3d). After hyperoxia, 60–90% of EVs carried caspase-3, as compared with the less than 20% of caspase-3-positive EVs detected in RA (Figure 3e). We next re-confirmed the origin of the caspase-3-positive EVs. Almost all of the caspase-3-positive EVs (99.5%) were derived from lung epithelial cells, which were positive on pan-cytokeratin staining (Figure 3f). To further confirm this observation, we first treated Beas2B cells with hyperoxia (95%). After 2 days' exposure, caspase-3 activity was robustly upregulated in these cells (Figure 3g). Using a variety of signaling inhibitors, we found that only an ATF6 inhibitor suppressed the hyperoxia-induced casepase-3 activity (Figure 3g). This inhibitory effect was further verified using ATF6 siRNA (Figure 3h).

### Hyperoxia-induced EVs mediated inflammatory lung responses *via* EV-shuttled caspase-3

**Figure 4:**
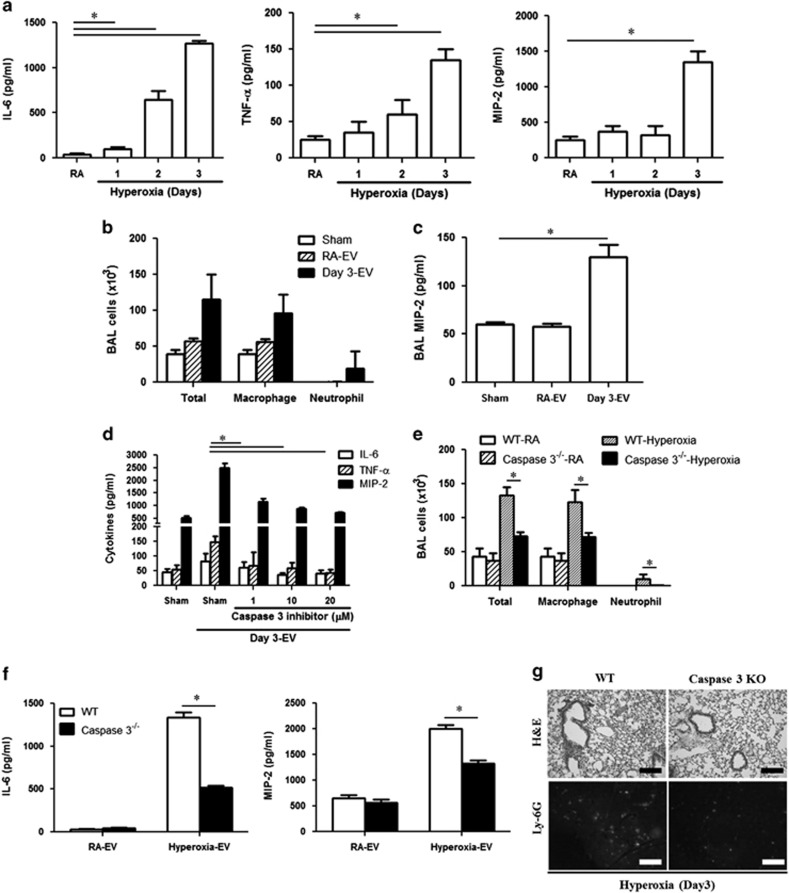
Pro-inflammatory effects of the EV-shuttled caspase-3. (**a**) EVs were isolated from mouse BALF after hyperoxia. Isolated primary alveolar macrophages were treated with 10 *μ*g/ml BALF EVs for 24 h. Cytokines were measured by ELISA. (**b**) Total and differential BALF cell counts were analyzed 24 h after delivering the RA-EVs and hyperoxia (3 days) induced EVs intranasally. (**c**) BALF MIP-2 was analyzed in the same condition as described in (**b**). (**d**) Cytokine production in MH-s was analyzed 24 h after treatment of EVs (10 *μ*g protein) in the presence of caspase-3 inhibitor. (**e**) BALF cell counts and differentials after hyperoxia (3 days) in WT or caspase-3-deficient mice. (**f**) BALF EVs were isolated from WT and caspase-3-deficient mice with or without hyperoxia (3 days). Isolated BALF EVs were used to treat primary alveolar macrophages for 24 h and cytokine production was measured using ELISA. (**g**) H&E-stained lung histology (upper) and Ly-6G-stained immunofluorescent image (lower) in mice after hyperoxia (3 days). Error bars represent mean±S.D. *n*=3. All other figures represent two independent experiments with identical results. *P<0.05

Activated alveolar macrophages are front-line immune cells, which are responsible for neutrophil recruitment *via* the release of cytokines/chemokines.^25^ We next evaluated the functions of the hyperoxia-induced, epithelial cell-derived EVs, using alveolar macrophages as the target cells. BALF EVs were isolated in mice exposed to RA or hyperoxia. After treating alveolar macrophages using these BALF EVs (10 *μ*g EV (protein)/sample), we found that hyperoxia-induced EVs stimulated the production of IL-6, TNF-*α* and MIP-2 (Figure 4a). Next, we isolated EVs from the supernatant of cultured primary alveolar type II cells after hyperoxia. When primary alveolar macrophages were treated with the type II epithelial cell-derived EVs, similar patterns of cytokine production were observed, as were observed in the macrophages treated with BALF EVs (Supplementary Figure S1D).

To test whether hyperoxia-induced EVs are responsible for propagating inflammatory lung responses after hyperoxia *in vivo*, we first isolated BALF EVs from mice exposed to RA or hyperoxia. Next, we delivered these EVs to wild type (WT) C57B/L6 mice intranasally. The BALF from these EV-treated mice was then obtained to analyze cell counts and cell differentials after 24 h. The BALF cell counts were significantly higher in the mice treated with the hyperoxia (3 days)-induced EVs (Figure 4b). The number of infiltrated alveolar macrophages and neutrophils were both augmented (Figure 4b). MIP-2 was also markedly increased in these BALF (Figure 4c). Interestingly, the pro-inflammatory effects of the hyperoxia-induced EVs were inhibited by caspase-3 inhibitors in a dose-dependent manner (Figure 4d). Bioactive recombinant caspase-3 stimulated the secretion of IL-6, TNF-*α* and MIP-2 in MH-s cells (Supplementary Figure S1E). Treating WT mice with the bioactive recombinant caspase-3 *via* inhalation resulted in a higher amount of BAL cells as well, but did not affect tissue damage (Supplementary Figure S1F and G). In addition, bioactive caspase-3 did not affect cell viability in lung epithelial cells (Beas2B, SAEC) or alveolar macrophages (MH-s) (Supplementary Figure S1H). To further confirm the importance of caspase-3 in hyperoxia-induced lung inflammation, we exposed the WT and caspase-3-deficient mice to 95% hyperoxia, as previously described.^20^ BALF was then obtained, cell counts were analyzed and EVs were isolated (Supplementary Figure S2A). Deletion of caspase-3 significantly decreased the macrophage and neutrophil infiltration in BALF (Figure 4e). Lung injury was reduced in caspase-3-deficient mice as well (Supplementary Figure S2B). Compared with WT EVs, caspase-3-deficient EVs markedly diminished the secretion of IL-6 and MIP-2 from alveolar macrophages (Figure 4f). Histologically, less cell infiltration was observed in caspase-3-deficient mouse lungs (Figure 4g). Ly-6G, a marker antigen of granulocytes and neutrophils, was significantly reduced in caspase-3-deficient mice after hyperoxia (3 days) (Figure 4g). Given that an elevated caspase-8 level was also detected in hyperoxia-induced EVs after 3 days' exposure (Figure 3b), we next evaluated whether caspase-8 alone or synergistically with caspase-3 exert pro-inflammatory effects on alveolar macrophages. We found that caspase-8 had no significant effect on IL-6, TNF-*α* or MIP-2 production, and there was no synergistic effect with caspase-3 (Supplementary Figure S2C).

### EV-shuttled caspase-3 induces ROCK1 expression and regulates macrophage functions via ROCK1-mediated pathways

**Figure 5:**
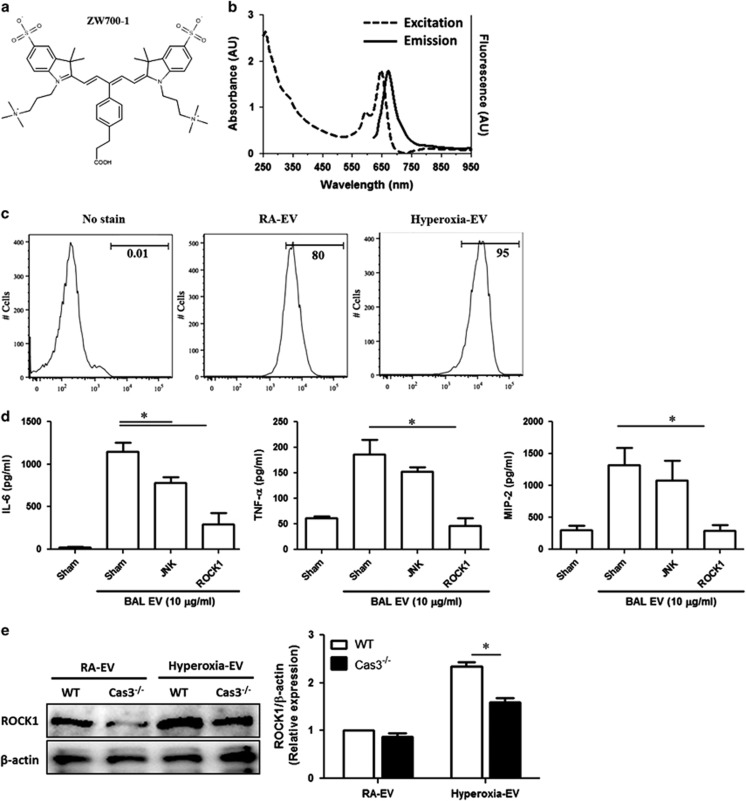
EV-shuttled caspase-3 augmented pro-inflammatory responses *via* ROCK1-mediated pathways in macrophages. (**a**) Chemical structure of ZW700-1 (Formular: C46H61N4O8S2, MW: 862.13, Log D at pH 7.4 :  −3.52). (**b**) Wavelength of excitation and emission of ZW700-1-conjugated EVs. (**c**) BAL EVs were isolated after RA and hyperoxia (3 days). Isolated EVs were conjugated with ZW700-1. These conjugated EVs were used to treat the alveolar macrophages for 2 h. Uptaken EVs were analyzed using a flow cytometer. (**d**) Hyperoxia-induced EVs were used to stimulate primary alveolar macrophages in the presence or absence of signaling inhibitors. Cytokines were measured in supernatant. (**e**) Expression of ROCK1 in the EVs isolated from the supernatant of the WT or caspase-3-deficient MH-s cells (left panel). Protein of ROCK1 was normalized using *β*-actin (right panel). Error bars represent mean±S.D. *n*=3. All other figures represent two independent experiments with identical results. *P<0.05

To investigate the mechanisms by which EV-shuttled caspase-3 activated alveolar macrophages, we isolated EVs from Beas2B cells 48 h after RA and hyperoxia exposure. Next, we labeled these isolated EVs with ZW700-1 (Figure 5a) and confirmed the wavelength of excitation and emission (Figure 5b). To elucidate the EV uptake in macrophages, we treated PMA-activated THP-1 cells with the ZW700-1 conjugated EVs. After 2 h, the EV uptake by macrophages was analyzed using flow cytometry. The ratios of EV uptake by macrophages were 95% in hyperoxia-induced EVs and 80% in RA-associated EVs (Figure 5c).

To determine the underlying mechanisms by which EV-shuttled caspase-3 activated alveolar macrophages, we pretreated the macrophages with signaling pathway inhibitors. Inhibitors of p38 MAPK, ROCK1 and JNK significantly blocked the caspase-3-induced IL-6 and TNF-*α* secretion in alveolar macrophages (Supplementary Figure S3A, left and middle panels). However, only ROCK1 and JNK inhibitors markedly reduced caspase-3-induced MIP-2 (Supplementary Figure S3A, right panel). Next, instead of using bioactive recombinant caspase-3, we treated the alveolar macrophages with the hyperoxia-induced EVs that were isolated from BALF, as described above. Interestingly, after being treated with hyperoxia-induced EVs, only the ROCK1 inhibitor, and not the JNK inhibitor, consistently suppressed IL-6, TNF-*α* and MIP-2 secretion in alveolar macrophages (Figure 5d). We found that recombinant caspase-3 rapidly upregulated the ROCK1 protein level in alveolar macrophages in a dose- and time-dependent manner (Supplementary Figure S3B and C). Consistently, ROCK1 was significantly upregulated in macrophages treated with the hyperoxia-induced EVs (Figure 5e). This effect was dramatically reduced when the macrophages were treated with caspase-3-deficient EVs, either in the absence or in the presence of hyperoxia (Figure 5e).

### Lung epithelial cell-derived, caspase-3-enriched EVs were detectable in serum and potentially activated systemic macrophages

**Figure 6:**
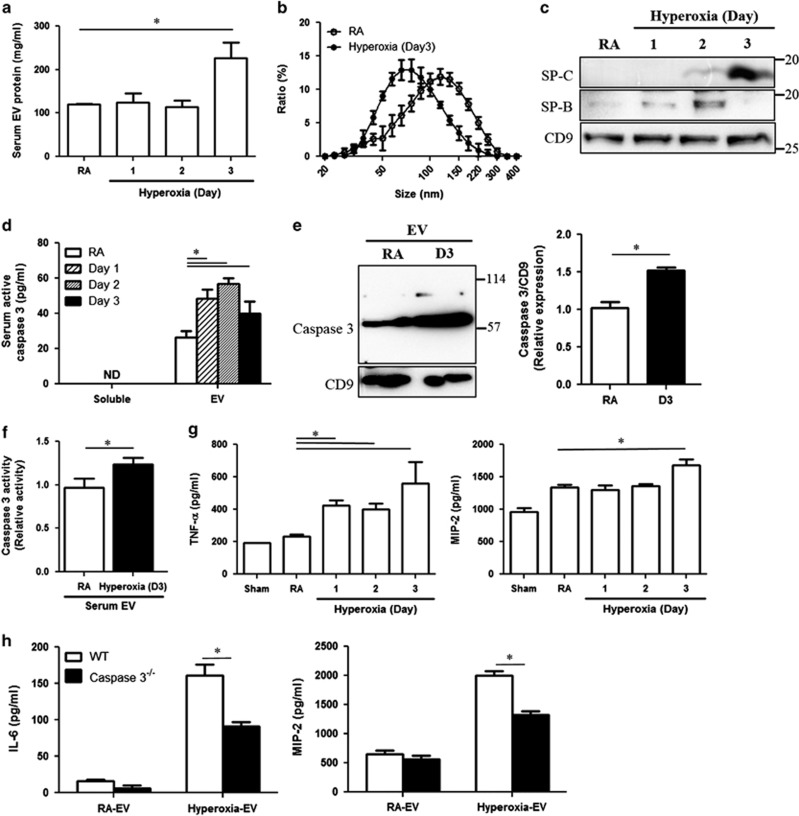
Analysis and characterization of serum EVs after hyperoxia. (**a**) Protein level of serum EVs after hyperoxia. (**b**) Size of serum EVs measured by DLS after 3 days' hyperoxia. (**c**) Expression of SP-B, SP-C, and CD9 in serum EVs. SP-B and SP-C are featured markers for lung epithelial cells. (**d**) Quantification of active caspase-3 in serum EVs and soluble fractions. (**e**) Expression of caspase-3 in serum EVs after hyperoxia (3 days). (**f**) Caspase-3 activity in serum EVs. (**g**) Hyperoxia-induced mouse serum EVs were used to treat peritoneal macrophages (J774). After 24 h, TNF-*α* and MIP-2 were analyzed using ELISA. (**h**) Serum EVs derived from the WT or caspase-3-deficient mouse were used to treat J774 macrophages. After 24 h, cytokine productions were determined. Error bars represent mean±S.D. *n*=3. All other figures represent two independent experiments with identical results. *P<0.05

To test whether the lung epithelial cell-derived caspase-3-enriched EVs enter the blood stream and thus potentially exert systemic effects, we exposed C57BL/6 mice to hyperoxia (95%). The EVs isolated from serum were significantly augmented after 3-day exposure to hyperoxia (Figure 6a), and the size was measured by DLS (Figure 6b). A time-dependent elevation of surfactant protein C (SP-C) and B (SP-B) was found in the serum EVs (Figure 6b). Given that SP-C and SP-B are alveolar epithelial cell marker proteins, these results suggest that these elevated serum EVs originate from epithelial cells. Active caspase-3 complex and activity were significantly induced in the serum EVs in a time-dependent manner after hyperoxia (Figures 6d–f). We next isolated the serum EVs from mice that had been exposed to RA or hyperoxia, as previously described.^20^ To determine whether the serum EVs activate macrophages in locations other than the lungs, the isolated serum EVs were used to treat peritoneal macrophages (J774 cells). After 24 h, elevated TNF-*α* and MIP-2 levels were detected in the peritoneal macrophages treated with hyperoxia-induced EVs, but not normoxia-associated ones (Figure 6f). Interestingly, deletion of caspase-3 using the EVs isolated from the hyperoxia-treated, caspase-3-deficient mice (caspase-3^−/−^) failed to induce TNF-*α* or MIP-2 secretion in the peritoneal macrophages (Figure 6h).

## Discussion

Lung inflammation is an essential feature in the pathogenesis of ALI/ARDS.^6^ In this report, we have proposed a novel paradigm that depends on the cross-talk between the lung epithelial cells and alveolar macrophages after oxidative stress. As shown in the schema (Supplementary Figure S4), hyperoxia stimulated the formation/release of lung epithelial cell-derived EVs. These epithelial cell-derived EVs in turn prompted a pro-inflammatory activation of macrophages, which subsequently resulted in neutrophil infiltration, inflammatory cytokine bursts and lung inflammation. We next discovered that EV-encapsulated caspase-3 plays a crucial role in mediating inflammatory lung responses. During hyperoxia induced DAD, a large amount of caspase-3 was produced that mediated the common pathway of oxidative stress-induced apoptosis.^22^ Thus, caspase-3-enriched EVs also mediate cross-talk between the cell death signaling pathways and the inflammatory signaling cascades.

The EVs that were derived from lung epithelial cells after hyperoxia fell into the size range of 50–120 nm, approximately the size of exosomes and microvesicles (Figure 1a). They are much smaller than apoptotic bodies, which range from 1 to 5 *μ*m.^10^ Hyperoxia-induced EVs expressed markers for both microvesicles and exosomes (Supplementary Figure S1A). As mentioned above, these epithelial cell-derived EVs were generated from live cells, not dying cells, further differentiating them from apoptotic bodies. Although the engulfing of apoptotic cells has been linked to inflammatory responses,^26, 27, 28^ our study provides evidence for a novel connection between the live cell-released EVs and targeted macrophage activation.

Previous reports have shown that epithelial cell death induces 'damage-associated molecular pattern molecules' (DAMPs) that initiate and perpetuate inflammation,^26, 27^ such as the nuclear or cytosolic proteins released from dying cells. DNA, mitochondrial DNA and RNA can all function as the DAMPs. Whether DAMPs are encapsulated into the EVs after certain stressful stimuli remains incompletely determined, but we failed to identify mitochondrial DNA in hyperoxia-induced EVs. Therefore, we focused mainly on the regulatory proteins that are known to mediate lung epithelial cell damage after hyperoxia, such as the caspases.

Caspase-1, one of the inflammasome components, regulates the inflammatory responses in macrophages after hyperoxia.^29, 30^ Pyroptosis, a caspase-1-mediated programmed cell death process that occurs after infectious insult, has also been reported to activate the inactive precursors of interleukin 1*β* and IL-18 into mature, inflammatory cytokines.^30^ Surprisingly, only a marginal increase in the caspase-1 level was detected in the BALF EVs obtained from the mice exposed to hyperoxia (3 days) (Figure 3). Our findings suggest that caspase-1 may not be responsible for the transmission of pro-inflammatory signals between epithelial cells and alveolar macrophages in the presence of oxidative stress. On the other hand, caspase-3, a central regulator for the common pathway of apoptosis, was enriched in the hyperoxia-induced, epithelial cell-derived EVs. Previous reports indicate that the generation of caspase-3-enriched membrane vesicles contributes to cellular homeostasis by the removal of intracellular caspase-3, and concurrently, protects the cells from direct exposure to caspase-3 activity and subsequent cell death.^31, 32^ Our study that in addition to the above-mentioned functions,^31^ caspase-3-enriched EVs may also transmit a ‘danger' or ‘pro-inflammatory' signal to the alveolar macrophages, subsequently triggering neutrophil influx and lung inflammation. These caspase-3-enriched EVs are largely released from live cells instead of apoptotic cells, serving as a ‘messenger' mediating cross-talk among live cells. The caspase-3 detected in the hyperoxia-induced EVs appeared to be the tetrameric form of caspase-3 (Figure 4). Upon stimuli, two copies of the large (p17) and small (p12) subunits comprising active caspase-3 are generated and form an active heterotetramer (MW: approximately 58 kD).^23, 24^ The tetrameric form of caspase-3 cleaves specific protein substrates within the cell, thereby resulting in apoptosis.^33^ There are only a few reports on the non-apoptotic functions of caspase-3, and these observations are in neurons and stem cell differentiation.^34, 35^ Böing *et al.* reported that MCF-7 breast cancer cells release caspase-3-containing vesicles, and these caspase-3-containing vesicles do not exhibit pro-apoptotic functions.^31^ Recently, emerging evidence has suggested that caspase-3 is detected in extracellular matrix (ECM), raising the hypothesis that caspase-3 participates in cell–cell communications in a paracrine manner.^36^ Our studies offer support to this hypothesis by demonstrating that caspase-3-enriched EVs facilitate certain macrophage-mediated pro-inflammatory effects. However, the sequestration of caspases can result in the tetrapeptide-based activity assays to underestimate the amount of caspase activation that has occurred *in situ*. In fact, this could well be happening in the case of EV-encapsulated caspase-3. Future studies will investigate in detail how caspase-3 folds in the EVs and whether it forms a multimer. Previously, EVs have been thought to bear similar cellular functions as their mother cells.^10, 18^ Our studies are among the very first reports to show that epithelial cell-derived EVs exert downstream functions on alveolar macrophages.

Mechanistically, caspase-3 regulated macrophage activation and pro-inflammatory cytokine release take place *via* Rho-associated Coiled-Coil kinase I (ROCK I) pathways (Figure 5). Previous reports have shown that ROCK I is cleaved by caspase-3 and subsequently contributes to membrane blebbing.^37, 38^ Although the role of membrane blebbing in cytokine release remains incompletely elucidated, it is very likely that the membrane blebbing induced by caspase-3-cleaved ROCK I is involved in Golgi-mediated exocytosis and cytokine release. Blocking ROCK I abolishes the effects of caspase-3 on pro-inflammatory cytokine release.

For a long time, ‘a messenger' between stressed lung epithelial cells and immunomodulatory cells has been suspected. Cytokines have been thought to pass these stress/inflammatory signals among cells.^18, 39, 40^ However, thus far, therapy targeting the inhibition of inflammatory cytokines has not been very effective for ARDS/ALI treatment.^41, 42, 43^ Furthermore, to fulfill the mission of a ‘signal messenger', a cytokine, as a protein, has to be resistant to proteinases in the ECM and has to efficiently enter target cells. The exosome probably serves as a kind of ‘shelter' that enables signaling proteins to avoid degradation in the ECM and thus serves as a cargo-carrier for transmitting a variety of ‘stress signals', including but not limited to caspase-3. One of the limitations in this investigation is that we do not address the entire protein content enwrapped in these epithelial cell-derived exosomes. A comprehensive proteomics on the epithelial cell-derived exosomes and the cell type-dependent EV production are planned in for the near future. Furthermore, the detailed signaling pathways of caspase-3-induced inflammatory responses in macrophages have not been fully determined. Identification of the caspase-3 conjugated receptor and signaling pathway, as well as the dose and time-dependent effects of exosome encapsulated caspase-3 on macrophage activation will also be taken up as a subject for further investigation in the near future.

In summary, this report provides a novel mechanism by which lung epithelial cells communicate with alveolar macrophages after exposure to devastating stimuli.

## Materials and Methods

Please refer to the online supplements for the details on the following sections:

Animals and *in vivo* hyperoxia exposure; Cell culture; *In vivo & in vitro* extracellular vesicle (EV) isolation; Extracellular vesicle surface staining; Sterile lung inflammation model; ELISA; Caspase-3 activity; Western blot; Statistical Analysis (ANOVA).

## Abbreviations

ALI: acute lung injury
ARDS: acute respiratory distress syndrome
BAL: bronchoalveolar lavage
DAD: diffuse alveolar damage
ER: endoplasmic reticulum
EV: extracellular vesicle
HALI: hyperoxia-induced ALI
MIP-2: macrophage inflammatory protein 2
MV: microvesicle
RA: room air
ROCK1: Rho-associated protein kinase 1
ROS: reactive oxygen species

## Supplementary Material

Supplementary Informations

## References

[bib1] 1Rubenfeld GD, Caldwell E, Peabody E, Weaver J, Martin DP, Neff M et al. Incidence and outcomes of acute lung injury. N Engl J Med 2005; 353: 1685–1693. 16236739 10.1056/NEJMoa050333

[bib2] 2Pagano A, Barazzone-Argiroffo C. Alveolar cell death in hyperoxia-induced lung injury. Ann NY Acad Sci 2003; 1010: 105–116. 10.1196/annals.1299.074 15033761

[bib3] 3Lamm WJ, Luchtel D, Albert RK. Sites of leakage in three models of acute lung injury. J Appl Physiol 1988; 64: 1079–1083. 3366730 10.1152/jappl.1988.64.3.1079

[bib4] 4Li X, Shu R, Filippatos G, Bhal BM. Apoptosis in lung injury and remodeling. J Appl Physiol 2004; 97: 1535–1542. 15358756 10.1152/japplphysiol.00519.2004

[bib5] 5Bhandari V. Molecular mechanisms of hyperoxia-induced acute lung injury. Front Biosci 2008; 13: 6653–6661. 18508685 10.2741/3179

[bib6] 6Matute-Bello G, Frevert CW, Martin TR. Animal models of acute lung injury. Am J Physiol Lung Cell Mol Physiol 2008; 295: L379–L399. 18621912 10.1152/ajplung.00010.2008 PMC2536793

[bib7] 7Jiang D, Liang J, Noble PW. Regulation of non-infectious lung injury, inflammation, and repair by the extracellular matrix glycosaminoglycan hyaluronan. Anat Rec (Hoboken) 2010; 293: 982–985. 20186964 10.1002/ar.21102 PMC2877145

[bib8] 8Matute-Bello G, Frevert CW, Martin TR. An official American Thoracic Society workshop report: features and measurements of experimental acute lung injury in animals. Am J Respir Cell Mol Biol 2011; 44: 725–738. 21531958 10.1165/rcmb.2009-0210ST PMC7328339

[bib9] 9Fukumoto J, Fukumoto I, Parthasarathy PT, Cox R, Huynh B, Ramanathan GK et al. NLRP3 deletion protects from hyperoxia-induced acute lung injury. Am J Physiol Cell Physiol 2013; 305: C182–C189. 23636457 10.1152/ajpcell.00086.2013 PMC3725631

[bib10] 10EL Andaloussi S, Mäger I, Breakefield XO, Woods MJ. Extracellular vesicles: biology and emerging therapeutic opportunities. Nat Rev Drug Discov 2013; 12: 347–357. 23584393 10.1038/nrd3978

[bib11] 11Li J, Liu K, Liu Y, Xu Y, Zhang F, Yang H et al. Exosomes mediate the cell-to-cell transmission of IFN-*α*-induced antiviral activity. Nat Immunol 2013; 14: 791–803. 10.1038/ni.2647 23832071

[bib12] 12Qin J, Xu Q. Functions and application of exosomes. Acta Pol Pharm 2014; 71: 537–543. 25272880

[bib13] 13Yoon YJ, Kim OY, Gho YS. Extracellular vesicles as emerging intercellular communicasomes. BMM Rep 2014; 17: 531–539. 10.5483/BMBRep.2014.47.10.164 PMC4261509 25104400

[bib14] 14Théry C, Ostrowski M, Segura E. Membrane vesicles as conveyors of immune responses. Nat Immunol 2009; 9: 581–593. 10.1038/nri2567 19498381

[bib15] 15Meckes Jr DG, Rab-Traub N. Microvesicles and viral infection. J Virol 2011; 85: 12844–12854. 21976651 10.1128/JVI.05853-11 PMC3233125

[bib16] 16Torrecilhas AC, Schumacher RI, Alves MJ, Colli W. Vesicles as carriers of virulence factors in parasitic protozoan diseases. Microbes Infect 2012; 14: 1465–1474. 22892602 10.1016/j.micinf.2012.07.008

[bib17] 17Hosseini HM, Fooladi AA, Nourani MR, Ghanezadeh F. The role of exosomes in infectious diseases. Inflamm Allergy Drug Targets 2013; 12: 29–37. 23441990 10.2174/1871528111312010005

[bib18] 18Keller S, Sanderson MP, Stoeck A, Alteveogt P. Exosomes: from biogenesis and secretion to biological function. Immunol Lett 2006; 107: 102–108. 17067686 10.1016/j.imlet.2006.09.005

[bib19] 19Bhatnagar S, Shinagawa K, Castellino FJ, Schorey JS. Exosomes released from macrophages infected with intracellular pathogens stimulate a proinflammatory response *in vitro* and *in vivo*. Blood 2007; 110: 3234–3244. 17666571 10.1182/blood-2007-03-079152 PMC2200902

[bib20] 20Zhang M, Lee SJ, An C, Xu JF, oshi B, Nabi IR et al. Caveolin-1 mediates Fas-BID signaling in hyperoxia-induced apoptosis. Free Radic Biol Med 2011; 50: 1252–1262. 21382479 10.1016/j.freeradbiomed.2011.02.031 PMC4134776

[bib21] 21Gewandter JS, Staversky RJ, O'Reilly MA. Hyperoxia augments ER-stress-induced cell death independent of BiP loss. Free Radic Biol Med 2009; 47: 1742–1752. 19786088 10.1016/j.freeradbiomed.2009.09.022 PMC2783969

[bib22] 22Mcllwain DR, Berger T, Mak TW. Caspase functions in cell death and disease. Cold Spring Harb Perspect Biol 2013; 5: a008659. 10.1101/cshperspect.a008656 PMC3683896 23545416

[bib23] 23Kang HJ, Lee YM, Jeong YJ, Park K, Jang M, Park SG et al. Large-scale preparation of active caspase-3 in E. coli by designing its thrombin-activatable precursors. BMC Biotechnol 2008; 8: 92. 19077216 10.1186/1472-6750-8-92 PMC2621203

[bib24] 24Ueta E, Kamatani T, Yamamoto T, Osaki T. Tyrosine-nitration of caspase 3 and cytochrome c does not suppress apoptosis induction in squamous cell carcinoma cells. Int J Cancer 2003; 103: 717–722. 12516089 10.1002/ijc.10832

[bib25] 25Hussell T, Bell TJ. Alveolar macrhopages: plasticity in a tissue-specific context. Nat Rev Immunol 2014; 14: 81–93. 24445666 10.1038/nri3600

[bib26] 26Kono H, Rock KL. How dying cells alert the immune system to danger. Nat Rev Immunol 2008; 8: 279–289. 18340345 10.1038/nri2215 PMC2763408

[bib27] 27Bellone M, Iezzi G, Rovere P, Galati G, Ronchetti A, Protti MP et al. Processing of engulfed apoptotic bodies yields T cell epitopes. J Immunol 1997; 159: 5391–5399. 9548479

[bib28] 28Berda-Haddad Y, Robert S, Salers P, Zekraoui L, Farnariere C, Dinarello CA et al. Sterile inflammation of endothelial cell-derived apoptotic bodies is mediated by interleukin-1*α*. Proc Natl Acad Sci USA 2011; 108: 20684–20689. 22143786 10.1073/pnas.1116848108 PMC3251090

[bib29] 29Rosin DL, Okusa MD. Dangers within: DAMP responses to damage and cell death in kidney disease. J Am Soc Nephrol 2011; 22: 416–425. 21335516 10.1681/ASN.2010040430 PMC4493973

[bib30] 30Tschopp J, Schroder K. NLRP3 inflammasome activation: The convergence of multiple signalling pathways on ROS production? Nat Rev Immunol 2010; 10: 210–215. 20168318 10.1038/nri2725

[bib31] 31Böing AN, Stap J, Hau CM, Afink GB, Ris-Stalpers C, Reits EA et al. Active caspase-3 is removed from cells by release of caspase-3-enriched vesicles. Biochim Biophys Acta 2013; 1833: 1844–1852. 23531593 10.1016/j.bbamcr.2013.03.013

[bib32] 32Loison F, Zhu H, Karatepe K, Kasorn A, Liu P, Ye K et al. Proteinase 3-dependent caspase-3 cleavage modulates neutrophil death and inflammation. J Clin Invest 2014; 124: 4445–4458. 25180606 10.1172/JCI76246 PMC4191030

[bib33] 33Fiandalo MV, Kyprianou N. Caspase control: protagonists of cancer cell apoptosis. Exp Oncol 2012; 34: 165–175. 23070001 PMC3721730

[bib34] 34Li Z, Sheng M. Caspases in synaptic plasticity. Mol Brain 2012; 14: 15. 10.1186/1756-6606-5-15 PMC3366905 22583788

[bib35] 35Janzen V, Fleming HE, Riedt T, Karlsson G, Riese MJ, Lo Celso C et al. Hematopoietic stem cell responsiveness to exogenous signals is limited by caspase-3. Cell Stem Cell 2008; 2: 584–594. 18522851 10.1016/j.stem.2008.03.012 PMC2991117

[bib36] 36Boland K, Flanagan L, Prehn JH. Paracrine control of tissue regeneration and cell proliferation by Caspase-3. Cell Death Dis 2013; 11: e725. 10.1038/cddis.2013.250 PMC3730423 23846227

[bib37] 37Gabet AS, Coulon S, Fricot A, Vandekerckhove J, Chang Y, Ribeil JA et al. Caspase-activated ROCK-1 allows erythroblast terminal maturation independently of cytokine-induced Rho signaling. Cell Death Differ 2011; 18: 678–689. 21072057 10.1038/cdd.2010.140 PMC3131901

[bib38] 38Coleman ML, Sahai EA, Yeo M, Bosch M, Dewar A, Olson MF. Membrane blebbing during apoptosis results from caspase-mediated activation of ROCK I. Nat Cell Biol 2001; 3: 339–345. 11283606 10.1038/35070009

[bib39] 39Hwang I. Cell-cell communication via extracellular membrane vesicles and its role in the immune response. Mol Cells 2013; 36: 105–111. 23807045 10.1007/s10059-013-0154-2 PMC3887950

[bib40] 40Turturici G, Tinnirello R, Sconzo G, Geraci F. Extracellular membrane vesicles as a mechanism of cell-to-cell communication: advantages and disadvantages. Am J Physiol Cell Physiol 2014; 306: C621–C633. 24452373 10.1152/ajpcell.00228.2013

[bib41] 41Lai KN, Leung JC, Metz CN, Lai FM, Bucala R, Lan HY. Role for macrophage migration inhibitory factor in acute respiratory distress syndrome. J Pathol 2003; 199: 496–508. 12635141 10.1002/path.1291

[bib42] 42Bertok S, Wilson MR, Morley PJ, de Wildt R, Bayliffe A, Takata M. Selective inhibition of intra-alveolar p55 TNF receptor attenuates ventilator-induced lung injury. Thorax 2012; 67: 244–251. 22156959 10.1136/thoraxjnl-2011-200590 PMC3282043

[bib43] 43Vadász I, Sznajder JI. Update in acute lung injury and critical care 2010. Am J Respir Crit Care Med 2011; 183: 1147–1152. 21531954 10.1164/rccm.201102-0327UP PMC3114050

