# Correction: Lung epithelial cell-derived extracellular vesicles activate macrophage-mediated inflammatory responses via ROCK1 pathway

**DOI:** 10.1038/s41419-020-2310-x

**Published:** 2020-02-12

**Authors:** H. -G. Moon, Y. Cao, J. Yang, J. H. Lee, H. S. Choi, Y. Jin

**Affiliations:** 1 Division of Pulmonary and Critical Care, Department of Medicine, Brigham and Women’s Hospital, Harvard Medical School, 75 Francis Street, Boston, MA 02115 USA; 2 Division of Hematology/Oncology, Department of Medicine, Beth Israel Deaconess Medical Center, Boston, MA 02215 USA

**Correction to: Cell Death and Disease**

10.1038/cddis.2015.282 published online 10 December 2015

This Article was originally published without the accompanying Supplementary figures. The Supplementary figures are provided below.

**Figure 1:**
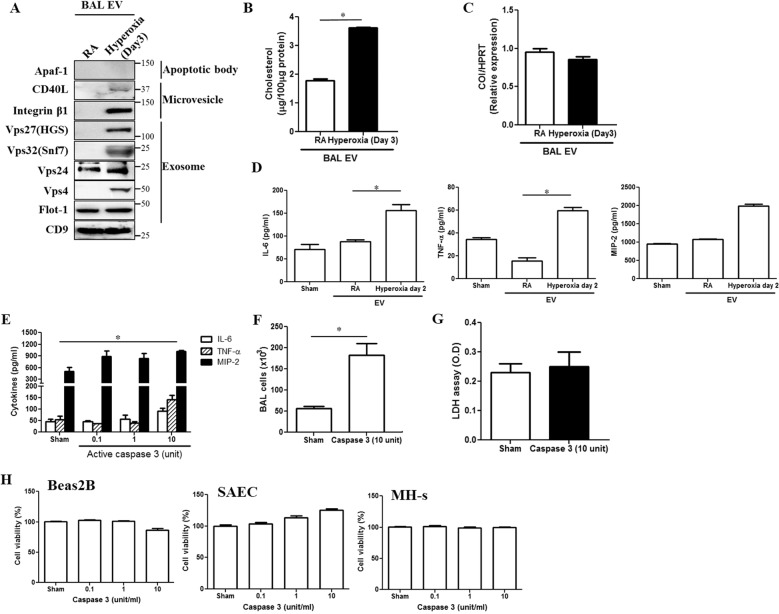
Supplement 1

**Figure 2:**
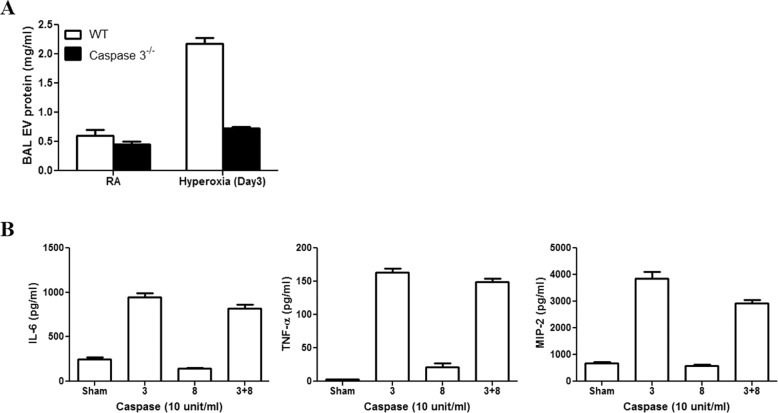
Supplement 2

**Figure 3:**
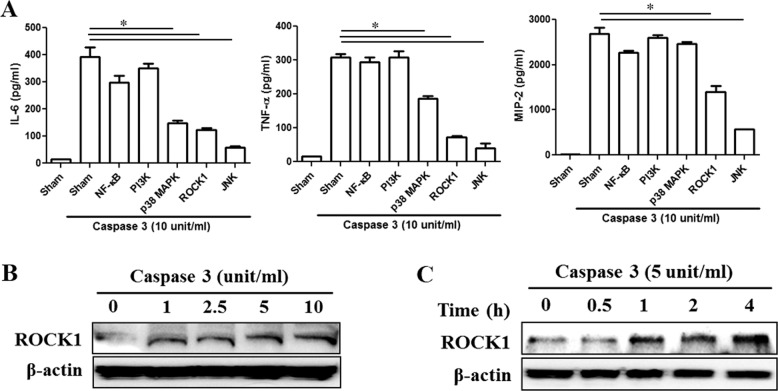
Supplement 3

**Figure 4:**
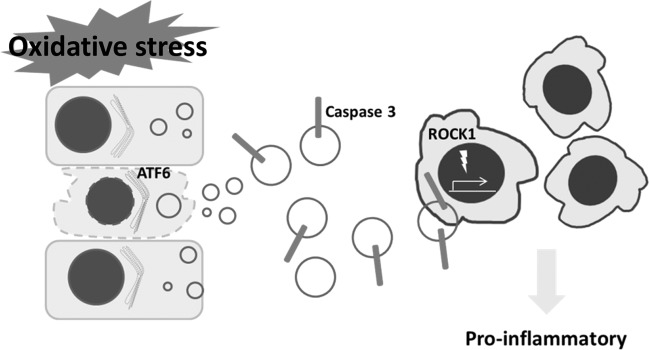
Supplement 4

