# Supplementary material for: Lung epithelial cell-derived extracellular vesicles activate macrophage-mediated inflammatory responses via ROCK1 pathway
Source: Cell Death Dis. 2015 Dec 10;6(12):e2016–. doi: 10.1038/cddis.2015.282 (PMC4720875; doi:10.1038/cddis.2015.282)
Supplement: Supplementary Informations [file cddis2015282x1.docx]

**On-line Supplements:**

**Supplement figure 1. Functional roles of recombinant caspase 3** (A) Confirmation of EV in BAL fluids with Apaf-1, CD40L, integrin β1, Vps24, Vps 32, Vps 24, Vps4 and flot-1 detection. (B) Cholesterol was measured in each 100 μg EVs by cholesterol assay kit. (C) Mitochondrial DNA (COI) was evaluated in BAL EV by real-time PCR. (D) MH-s cells were treated with 10 μg hyperoxia-induced, primary type II lung epithelial cell-derived EVs, for 24h. All cytokines were measured using ELISA. (E) Cytokine production after recombinant caspase-3 treatment in MH-s for 24h. (F) BALF cell counts were analyzed after recombinant caspase-3 (10 unit) administered intranasally. (G) LDH was measured in the same experimental condition with (F). (H) Cell viability of Beas-2B (left), small airway epithelial cells (SAECs) (middle) and alveolar macrophages (right) cells after exposure to the bioactive, recombinant caspase-3. Error bars represent Mean +/- SD. n=3. All other figures represent two independent experiments with identical results.*p<.05

**Supplement 2. The level of EVs in the WT or Caspase-3-deficient mice after hyperoxia (3 d).** (A) Protein concentration of BALF EVs (B) No synergetic effects of caspase-3 and caspase-8. Bioactive recombinant caspase-3 and -8 were used to treat MH-s cells. After 24h, synergetic effects were evaluated by measuring cytokines in supernatant. Error bars represent Mean +/- SD. n=3. *p<.05

**Supplement 3.** **Pro-inflammatory effects of bioactive recombinant caspase-3** (A) MH-s cells were treated with recombinant caspase-3 (10 u/ml) for 24h in the presence or absence of signaling inhibitors. Each inhibitor (10 μM) was delivered 1h prior to the administration of caspase-3. Cytokines were measured in supernatant. (B) Recombinant caspase-3 induced ROCK1 expression in MH-s cells in a dose- and (C) time-dependent manner. Error bars represent Mean +/- SD. n=3. All other figures represent two independent experiments with identical results.*p<.05

**Supplement 4. Schemata:** Hyperoxia stimulated the formation and release of lung epithelial cell-derived EVs *via* ER stress. Active caspase-3 was encapsulated into EVs and took the role of intercellular communicator between lung epithelial cells and alveolar macrophages. EV enriched caspase-3 stimulated alveolar macrophages *via* ROCK1 pathway and augmented the secretion of pro-inflammatory cytokines.
